# Individualization

**Published:** 1894-05

**Authors:** 


THE
### HOMŒOPATHIC PHYSICIAN,
A MONTHLY JOURNAL OF
HOMŒOPATHIC MATERIA MEDICA AND CLINICAL MEDICINE.

"If our school ever gives up the strict inductive method of Hahnemann, we are lost, and deserve only to be mentioned as a caricature in the history of medicine."—CONSTANTINE HERING.

Vol. XIV. MAY, 1894. No. 5.

### EDITORIAL.

INDIVIDUALIZATION.—In the editorial of last month we pointed out the effects of generalization in influencing the acceptance of homœopathic doctrine.

The difficulty created by this habit of mind is still further increased by the exactions of the practical employment of the homœopathic method in particular cases in the daily life of the physician. Instead of his being able to give the same remedy again and again in what superficially appear to be similar cases, and thus reducing his practice to a routine that saves him from much mental effort and loss of time, and consequent interference with his personal ease and comfort, he finds that every case is a new study. That the remedies and experience acquired in one case are of no use in another, and that the case must be studied by itself and without reference to its predecessors. The indications that led him to a successful prescription in one case do not occur in the next one, and other and possibly unfamiliar symptoms crop out instead. This compels a perusal of the repertories and a close study of the materia medica, with consequent loss of time, perplexity of mind as to getting the totality of the symptoms, and the additional mental tax involved in a new method of thinking—that of particularizing, or of *individualization* brought into opposition to the ingrained habit of generalization as previously explained in the April editorial.

It should not, therefore, be surprising that a man who has so far overcome his skepticism as to enter upon the practice of Homœopathy should draw back from the task that confronts him.

Such a man will be found unconsciously seeking short-cut methods of applying the law of the similars, and failing that, using methods outside of the principle, and which he calls "adjuvants." Thus arises eclecticism in the homœopathic school with its host of strenuous advocates.

Physicians professing to practice Homœopathy do not sufficiently realize that any combination of symptoms known in the category of human suffering may be found in any particular case of any given disease. These combinations are varied and unexpected to the last degree; making of the human system a veritable kaleidoscope, in which the most remarkable patterns occur, in infinite variety.

To meet these combinations of symptoms the practitioner must have ready a vast knowledge of the most diverse symptoms of the materia medica, so that he may promptly cover the combination with the truly similar remedy. How many of us are so equipped? Scarcely one. The faithful struggle along under the burden, making wonderful cures here, producing unsatisfactory palliation there, and failing totally elsewhere, but always stimulated to attain the ideal cure by the encouragement which they get from the undoubted successes they have achieved.

As for the unfaithful, they abandon the whole thing in disgust, do not try to find the simillimum, and join the ranks of the eclectics, declaring the whole fabric a failure, or that *similia* is only a rule which is sometimes useful.

These form the majority. Their desertion of the cause keeps the truth yet a longer time in oblivion.

What, then, shall be said in praise of that little band of workers who, vividly realizing the task before them, and undismayed by the extent of it, resolutely start out to climb the Hill of Difficulty and bring the truth up with them into the clear light of Universal Knowledge?

